# Mapping Critical Language Sites in Children Performing Verb Generation: Whole-Brain Connectivity and Graph Theoretical Analysis in MEG

**DOI:** 10.3389/fnhum.2017.00173

**Published:** 2017-04-05

**Authors:** Vahab Youssofzadeh, Brady J. Williamson, Darren S. Kadis

**Affiliations:** ^1^Pediatric Neuroimaging Research Consortium (PNRC), Cincinnati Children’s Hospital Medical CenterCincinnati, OH, USA; ^2^Division of Neurology, Cincinnati Children’s Hospital Medical CenterCincinnati, OH, USA; ^3^Department of Psychology, University of CincinnatiCincinnati, OH, USA; ^4^College of Medicine, Department of Pediatrics, University of CincinnatiCincinnati, OH, USA

**Keywords:** magnetoencephalography, hubs, eigenvector centrality, expressive language, verb generation, graph theory, connectivity, phase locking value

## Abstract

A classic left frontal-temporal brain network is known to support language processes. However, the level of participation of constituent regions, and the contribution of extra-canonical areas, is not fully understood; this is particularly true in children, and in individuals who have experienced early neurological insult. In the present work, we propose whole-brain connectivity and graph-theoretical analysis of magnetoencephalography (MEG) source estimates to provide robust maps of the pediatric expressive language network. We examined neuromagnetic data from a group of typically-developing young children (*n* = 15, ages 4–6 years) and adolescents (*n* = 14, 16–18 years) completing an auditory verb generation task in MEG. All source analyses were carried out using a linearly-constrained minimum-variance (LCMV) beamformer. Conventional differential analyses revealed significant (*p* < 0.05, corrected) low-beta (13–23 Hz) event related desynchrony (ERD) focused in the left inferior frontal region (Broca’s area) in both groups, consistent with previous studies. Connectivity analyses were carried out in broadband (3–30 Hz) on time-course estimates obtained at the voxel level. Patterns of connectivity were characterized by *phase locking value* (PLV), and network hubs identified through *eigenvector centrality (EVC)*. Hub analysis revealed the importance of left perisylvian sites, i.e., Broca’s and Wernicke’s areas, across groups. The hemispheric distribution of frontal and temporal lobe EVC values was asymmetrical in most subjects; left dominant EVC was observed in 20% of young children, and 71% of adolescents. Interestingly, the adolescent group demonstrated increased critical sites in the right cerebellum, left inferior frontal gyrus (IFG) and left putamen. Here, we show that whole brain connectivity and network analysis can be used to map critical language sites in typical development; these methods may be useful for defining the margins of eloquent tissue in neurosurgical candidates.

## Introduction

Neuroimaging studies using functional magnetic resonance imaging (fMRI) and magnetoencephalography (MEG) have consistently identified a left-lateralized frontal-temporal functional network for language processing in the vast majority of right-handed adults (~95%; Binder et al., 1997; Gabrieli et al., 1998; Price, 2000; Hirata et al., 2004; Lohmann et al., 2010; Friederici, 2011, 2012; Kadis et al., 2011; Pei et al., 2011; Turken and Dronkers, 2011). The network includes Broca’s area, in the posterior convolutions of the left inferior frontal gyrus (IFG; see Broca, 1861), and Wernicke’s area in the posterior left superior temporal gyrus (STG; Wernicke, 1874) responsible for *expressive* and *receptive* language processes, respectively. Developmental language studies have reported similar frontal-temporal network for children, but with decreased lateralization and increased engagement beyond the canonical language network (Holland et al., 2007; Karunanayaka et al., 2007; Kadis et al., 2011; Berl et al., 2014). Active involvement of subcortical and cerebellar regions have also been reported in imaging studies (Frings et al., 2006; Booth et al., 2007; Houk et al., 2007; Murdoch, 2010; Berl et al., 2014; Verly et al., 2014). However, the level of participation of constituent regions, and the contribution of extra-canonical areas, is not fully understood; this is particularly true for pediatrics, and in individuals who have experienced early neurological insult.

More recently, researchers have begun to use sophisticated analytic strategies such as functional and effective connectivity analysis to explore regional interactions for language (Bitan et al., 2005, 2006; Sonty et al., 2007; Allen et al., 2008; Leff et al., 2008; Schofield et al., 2009; David et al., 2011; Doesburg et al., 2012, 2015; Verly et al., 2014; Kadis et al., 2015; Xiao et al., 2016). In our recent MEG study of verb generation in children, we observed frequency dependent patterns of connectivity together with an increased number of suprathreshold effective (directed) connections with age, even though the extent of the network decreased and became increasingly left lateralized with age (Kadis et al., 2015). Others have used dynamic causal modeling (DCM) in fMRI to show that higher level language processing is relatively left lateralized, compared to basic sensory processes (Bitan et al., 2005, 2006; Sonty et al., 2007; Allen et al., 2008; Cao et al., 2008; Leff et al., 2008; Schofield et al., 2009; Xiao et al., 2016). In addition, involvement of subcortical structures in language processing has been supported by DCM for MEG and fMRI data related to auditory comprehension task (Booth et al., 2007; David et al., 2011). However, these studies have mainly focused on interactions of distinct nodal regions (seeds) or limited prespecified regions of interest (ROIs). In the case of DCM, a clear hypothesis regarding the directionality of coupling and network architecture is also required. As a result, the complexity of the brain network controlling language has remained largely unresolved with neuroimaging.

Graph theoretical measures are increasingly used to reveal non-trivial characteristics of human brain networks, including functional integration (summary statistics of broad connectivity patterns) and segregation (the identification of specialized regions or local groups; Rubinov and Sporns, 2010). The importance of a particular node may be determined by the amount or quality of its connectedness (where highly connection nodes are termed “hubs”; He and Evans, 2010; McIntosh and Mišić, 2013; Power et al., 2013; Fornito et al., 2015; Fuertinger et al., 2015). Investigating hubs can help to answer questions of how complex networks might develop, or how networks are altered in neuropsychiatric disease, such as Alzheimer’s or schizophrenia (Achard and Bullmore, 2007; Bassett et al., 2009; Stam et al., 2009; Supekar et al., 2009; Mandelli et al., 2016).

Here, we propose whole-brain connectivity and graph-theoretical analysis of task-related MEG source estimates to provide robust maps of the pediatric expressive language network. We quantify voxel-level connectivity using the *phase locking value* (PLV) metric (Lachaux et al., 1999) and network hubs via three graph metrics: *degree, eigenvector centrality (EVC)* and* betweenness centrality* (Rubinov and Sporns, 2010). We employ automated parcellation to summarize voxel-level connectivity findings as they relate to age.

## Materials and Methods

### Participants and Experiment Design

Two groups of subjects were recruited for this study: 15 typically-developing young children (8 females, aged 4–6 years, mean ± SD: 5.6 ± 0.94) and 14 adolescents (8 females, aged 16–18, 17.18 ± 0.79). Participants were recruited from the community (Cincinnati), through flyers and online advertisements. All were native English speakers without history of neurological insult, speech or language disorder, or learning disability; all scanning was conducted for the sole purpose of research participation. This study was carried out in accordance with the recommendations of title 45, part 46, and title 21 parts 50 and 56, of the Code of Federal Regulations, with written informed consent from all subjects. All subjects gave written informed consent or parental consent and child assent were obtained in all cases in accordance with the Declaration of Helsinki. The study was approved by the Institutional Review Board (IRB) at Cincinnati Children’s Hospital Medical Center. Participants received compensation for travel and participation.

Subjects completed a covert auditory verb generation MEG experiment. Target stimuli were auditorily presented and subjects were instructed to rapidly think of an action word that corresponded to the presented noun. Items were chosen from normative databases and standardized language assessments; each item and its usage were familiar to typically-developing 5-year old children, (e.g., book, dog, pencil). Development of the stimulus set has been described previously (Kadis et al., 2011). Prior to the MEG recording, participants were trained on an overt version of the task in order to establish sufficient ability and promote compliance during subsequent acquisition.

The control task involved passive listening to noise stimuli (contoured noise, matched to target stimuli in terms of spectral content and amplitude envelope). Target and noise stimuli were identical in duration (0.67 ± 0.14 s), presented alternately, once every 4–5 s (range reflects random jitter). A total of 71 distinct nouns and 71 noise trials were presented. Since we were interested in verb generation (expressive language), we focused on responses to nouns only.

### Data Acquisition

Participants underwent MEG, MRI, fMRI and diffusion imaging in this study; only MEG and structural MRI are analyzed, here. MEG data were acquired using a whole-head 275-channel CTF system (MEG International Services Ltd., Coquitlam, BC, Canada) with a sampling rate of 1.2 kHz. All participants were tested in a supine position with their heads supported on memory foam and linens for comfort and stability. We limited the duration of scanning to less than 20 min of acquisition (total, across all MEG paradigms), to minimize fatigue and disinterest. Stimuli were presented via a calibrated audio system comprised of a distal transducer, tubing and ear inserts (Etymotic Research, IL, USA). Head localization coils were placed at nasion and preauricular points to monitor movement. In all cases, head displacement was less than 5 mm from the beginning to end of acquisition. To facilitate MEG coregistration, multimodal radiographic markers were placed at the same fiducial locations before acquiring structural images.

MRI was conducted at 3.0T on a Philips Achieva scanner (Philips Medical Systems) located at Cincinnati Children’s Hospital Medical Center. A whole brain 3D T1-weighted image was acquired using an MDEFT scan (flip angle = 90°, TE = 3.7 ms, TR = 8.1 ms, TI = 939 ms, voxel size = 1.0 × 1.0 × 1.0 mm).

### Data Analysis

Data were processed using FieldTrip toolbox (Oostenveld et al., 2011) and graph theoretical analyses were carried out using the Brain Connectivity Toolbox (Rubinov and Sporns, 2010) in MATLAB R2016a (The Mathworks, Inc., Natick, MA, USA).

#### Preprocessing and Response-Locked Analysis

The raw data were initially epoched from −900 ms to 1800 ms relative to the onset of stimuli, and baseline corrected using the −900 ms to 0 ms window. Line noise was attenuated at 60, 120, 180 Hz by means of a very sharp discrete Fourier transform filter, and bandpass filtered from 0.1 Hz to 100 Hz. Scanner jump artifacts were automatically identified (by means of a median filter) and rejected from trials. An average of 3.1 ± 3.8 (mean ± STD) trials were rejected across individuals. To minimize edge effects, we selected a wide initial window, free from overlap across trials/conditions. The baseline and active windows were subsequently defined as −400 ms to 0 ms and 600–1000 ms, respectively. In previous verb generation studies, researchers have documented strong beta-band *event related desynchrony* (ERD) ~300–800 ms following picture presentation (Kadis et al., 2008, 2011; Ressel et al., 2008; Pang et al., 2011). However, the use of audio stimuli in the current study necessitates focusing on a later, active window (to account for the delay we shifted the “active” window out by 300 ms).

#### Source Inversion

The covariance matrix, a key ingredient for source inversion, was computed from whole window (combined data epochs of [−400 to 0] and [600–1000] ms), so called “common filter” for each subject and each condition. The common filter improves the source estimation (see Wibral et al., 2011; Haegens et al., 2014). Anatomical MR scans of subjects were segmented and tissue types (brain, skull and scalp surface) were extracted. MEG and MRI scans were coregistered using the common fiducial markers. Single shell head models were constructed from the segmented MRI (Nolte, 2003). A 3-D grid was constructed with a dipole resolution of 10 mm (spacing inside the brain), and the lead field computed for each grid point. To estimate time-series sources, a linearly constrained minimum-variance (LCMV) beamformer with 0.1% regularization was used (Van Veen et al., 1997). A differential beamformer analysis (mean power difference between active and baseline windows) was performed. We report source activations in the low-beta (13–23 Hz) band, to be consistent with literature (Kadis et al., 2008, 2011; Ressel et al., 2008; Pang et al., 2011). We performed a Monte Carlo test with 5000 randomizations, to identify significant (*p* < 0.05) low-beta ERD for each individual. Statistics for each random reshuffling was computed by a paired/dependent-sample *t-test* and multiple comparisons were corrected with an FDR threshold value of *q* = 0.05. For visualization, we spatially normalized sources to a template brain in MNI space. The scaling of child and adolescent brains into adult template space is generally considered minimal (for example see Burgund et al., 2002).

#### Connectivity Analysis

For connectivity analysis, time-series sources were estimated in a broadband (3–30 Hz) frequency range. Whole-brain connectivity patterns were assessed by computing the PLV for each voxel pair during both “active” (600–1000 ms following stimulus onset) and “baseline” (−400 ms to 0 ms from stimulus onset) periods (Lachaux et al., 1999): $\text{PLV}(\omega )\;=\;\left|{\langle \sum^{{\text{e}}^{j\emptyset (\omega )}}\rangle }_{N}\right|$ where the phase difference ∅(ω) was derived from Fourier transform of voxel timeseries across *N* trials. This metric was selected since we were interested in characterizing network maps based on the presence of connections, independent of the directionality of connections. We computed active minus the baseline window connectivity to isolate task-related effects.

#### Network Analysis

A network analysis from the graph theory perspective was performed on the adjacency matrices. The adjacency matrices were binarized based on an arbitrary threshold of 0.7, corresponding to 70% maximum connectivity strength for each subject. Comparison of three arbitrary thresholds of 50, 70 and 90% applied to a network by EVC measure is shown in Supplementary Figure S1. To identify hubs, we computed three measures of centrality, including *degree*, *EVC* and *betweenness centrality*, as implemented in Brain Connectivity Toolbox (Rubinov and Sporns, 2010). Most simply, network degree is the total number of suprathreshold connections at each node, EVC is an extension of degree (it assigns relative scores to the nodes based on their neighbors’ degrees), and betweenness centrality is a tendency for a node to occupy positions along shortest paths (Freeman, 1977; Bonacich, 2007).

For a group analysis, an automated anatomical labeling (AAL) atlas (Tzourio-Mazoyer et al., 2002) consisting of 116 subdivisions of cortical, subcortical and cerebellar regions was used to combine mean network measures computed at voxel-level in each parcel. Group differences in network findings (i.e., hub strength) were assessed using an independent samples *t*-test (FDR with *q* = 0.05).

To characterize hemispheric involvement, we derived a conventional laterality index, LI = (L − R)/(L + R), for the EVC scores within all frontal and temporal lobe parcels (15 distinct anatomical regions per hemisphere). LI values greater than 0.25 were considered as left-lateralized, less than −0.25 as right-lateralized, and intermediate values as a bilateral.

## Results

**Figure 1:**
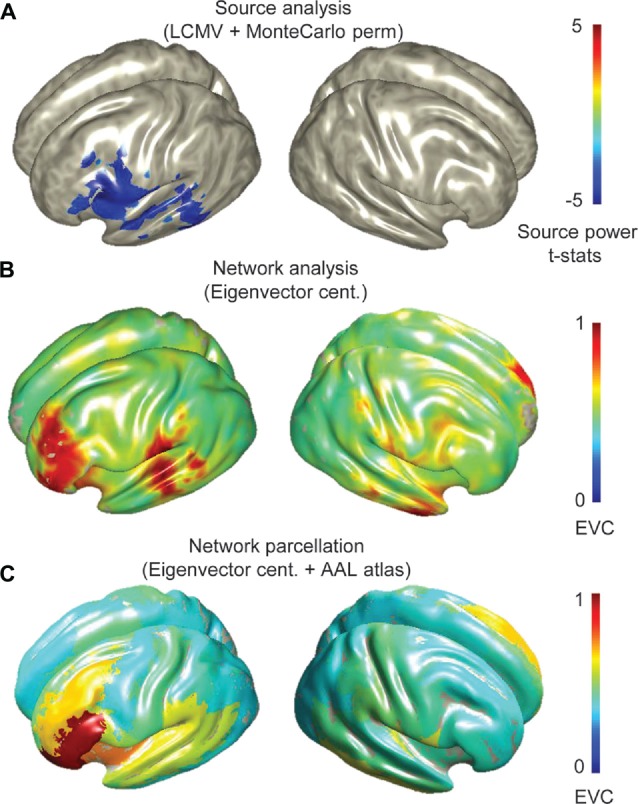
**Group source and network analysis of all participants (14 adolescents and 15 children) during verb generation magnetoencephalography (MEG) experiment. (A)** Topographical map of grand averaged source activations from differential beamformer analyses and statistically validated by Monte Carlo simulations (permutation test with an alpha level of 0.05, 5000 randomizations and FDR correction with *q* = 0.05 for multiple comparisons), showing beta event related desynchrony (ERD) in perisylvian regions. **(B)** Network maps captured by eigenvector centrality (EVC) at the voxel-level, and **(C)** parcellated EVC. Group average network measures were scaled between 0 and 1.

The groups averaged sources revealed prominent beta (13–30 Hz) ERD in inferior frontal and posterior temporal regions i.e., Broca’s and Wernicke’s regions (Figure 1A). Voxel-wise and parcellated network maps indicate the importance of left perisylvian sites for language expression in both groups (Figures 1B,C, see Supplementary Figure S2 for parcellated maps of individuals).

**Figure 2:**
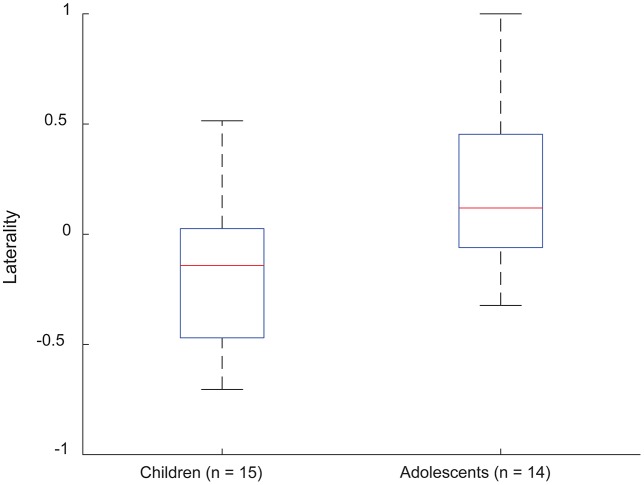
**Laterality indices for EVC in frontotemporal parcels, for young children and adolescents performing verb generation.** Positivity and negativity correspond to left- and right-lateralized EVC distributions, respectively.

Laterality indices based on EVC suggested a left-hemispheric dominance in 3/15 (20%) young children and 10/14 (71%) adolescents. Other subjects were either bilateral (10/15, or 66.6% of young children, and 4/14, or 28% of adolescents) or had right-hemisphere dominance (2/15, or 13.3% of young children). A boxplot of LI variations is shown in Figure 2, indicating a range of [−0.1952, 0.1428] and [−0.0896, 0.2773] for the young children and adolescents, respectively.

**Figure 3:**
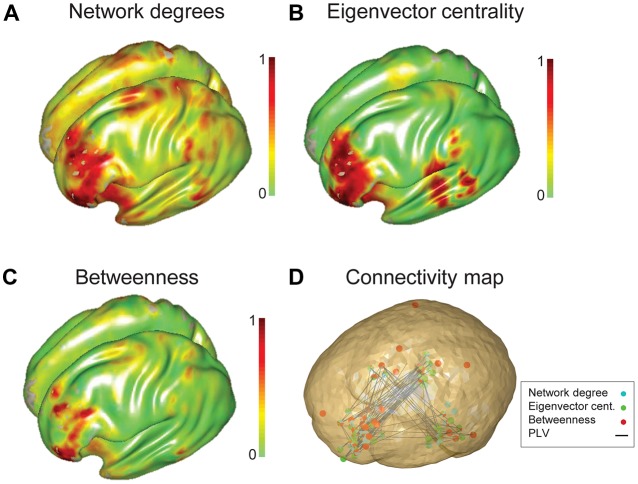
**Group network analysis of all participants characterized using three graph theory measures.** Three graph theory measures, **(A)** network degree, **(B)** EVC and **(C)** betweenness centrality were used to characterize the language network of all subjects (15 adolescents and 15 children). **(D)** Connectivity map overlaid by thresholded (10% of nodes with the highest level of eigenvalue centrality) three network measures. Nodes captured by degree centrality, EVC and betweenness centrality values are specified by cyan, green and red filled circles.

Network maps constructed from each of the centrality measures were generally consistent, showing predominant hubs in left prefrontal cortex. The pattern of hub distribution for degree and EVC were nearly identical; in contrast, the map derived from betweenness centrality was relatively focal to the left IFG (Figure 3). Visual analyses suggested a better consistency for EVC (than the other two measures) with the connectivity patterns demonstrated on a subject-wise basis; hence EVC was preferred for subsequent analyses and interpretations.

**Figure 4:**
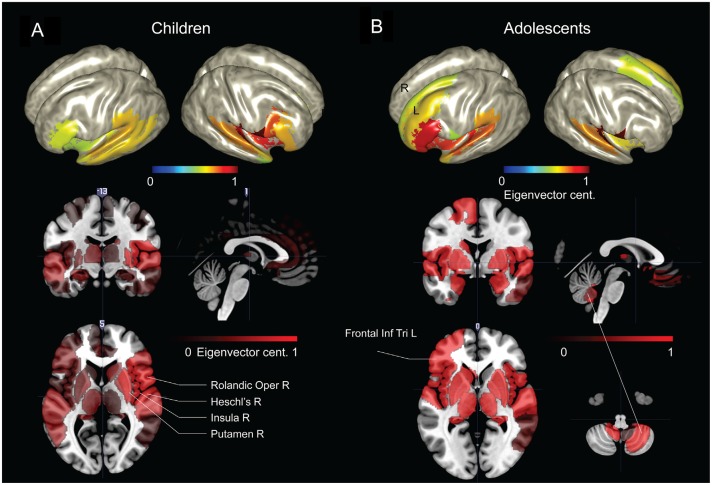
**Group network-based parcellation of adolescents and children. (A)** Cortical and subcortical regions detected during group network analysis through EVC from children and **(B)** adolescents. Results have been threshold with an arbitrary value of 0.7.

The parcellated maps for the group of young children suggested stronger hubs in right, rather than left hemisphere regions (Figure 4A). As summarized in Table 1, the EVC values for young children in the order of strength were, right Rolandic operculum, right Heschl’s gyrus, right insula, right putamen, right inferior frontal operculum, right STG, left pars orbitalis, right pars triangularis and left STG. In comparison, group parcellated map of adolescents showed strong hubs in left perisylvian regions, right Heschl’s gyrus (or primary auditory cortex) in STG area and bilateral subcortical regions (Figure 4B). Important ROIs in the order of network strength were, right Rolandic operculum, right Heschl’s gyrus, left amygdala, left globus pallidus, left putamen, left pars triangularis, left Heschl’s gyrus, left pars orbitalis, left insula and left STG. Unlike the adolescent group, stronger hubs were found in right cortical and subcortical regions of children, as in Figure 4A and Table 1. This may support the hypothesis that right hemisphere supports language in very early childhood (Chiron et al., 1997; see “Discussion” Section). We also observed hubs in cerebellar regions of adolescents and not children, mainly at right cerebellar hemisphere (see Figure 4, not reported in Table 1). Overall, hubs identified for children were stronger in right frontotemporal cortical and subcortical regions, but weaker at the left prefrontal and cerebellar regions.

**Table 1:** **Summary of regions of interest (ROIs) detected by network-based parcellated maps of two groups of subjects, adolescents and children**.

Children		Adolescents		Adolescents—Children		Children—Adolescents	
ROI	EVC (mean ± SE)	ROI	EVC (mean ± SE)	ROI	P (FDR)	ROI	P (FDR)
Rolandic Oper R	0.6 ± 0.02	Rolandic Oper R	0.56 ± 0.01	**Cerebellum 10 R**	0.0097	**Frontal Inf Tri R**	0.0026
Heschl R	0.56 ± 0.02	Heschl R	0.56 ± 0.01	**Cerebellum 8 R**	0.004	**Caudate R**	0.0037
Insula R	0.48 ± 0.01	Amygdala L	0.54 ± 0.02	**Frontal Inf Orb L**	0.045	**Frontal Inf Orb R**	0.088
Putamen R	0.47 ± 0.01	Pallidum L	0.52 ± 0.02	**Cerebellum 7b R**	0.0011	**Frontal Inf Oper R**	0.036
Frontal Inf Oper R	0.46 ± 0.01	Putamen L	0.51 ± 0.02	**Cerebellum 3 L**	0.028	**Olfactory R**	0.036
Temporal Sup R	0.43 ± 0.01	Frontal Inf Tri L	0.5 ± 0.02	Frontal Med Orb L	0.22	**Precentral L**	0.032
Frontal Inf Orb R	0.41 ± 0.02	Heschl L	0.47 ± 0.01	Putamen L	0.060	**Temporal Pole R**	0.030
Pallidum L	0.41 ± 0.02	Frontal Inf Orb L	0.45 ± 0.02	Amygdala L	0.23	**Cingulum Ant L**	0.026
Frontal Inf Tri R	0.41 ± 0.02	Insula L	0.45 ± 0.01	Frontal Inf Tri L	0.33	Insula R	0.060
Temporal Sup L	0.4 ± 0.01	Temporal Sup L	0.42 ± 0.01	Vermis 9	0.11	Cingulum Ant R	0.06

**Figure 5:**
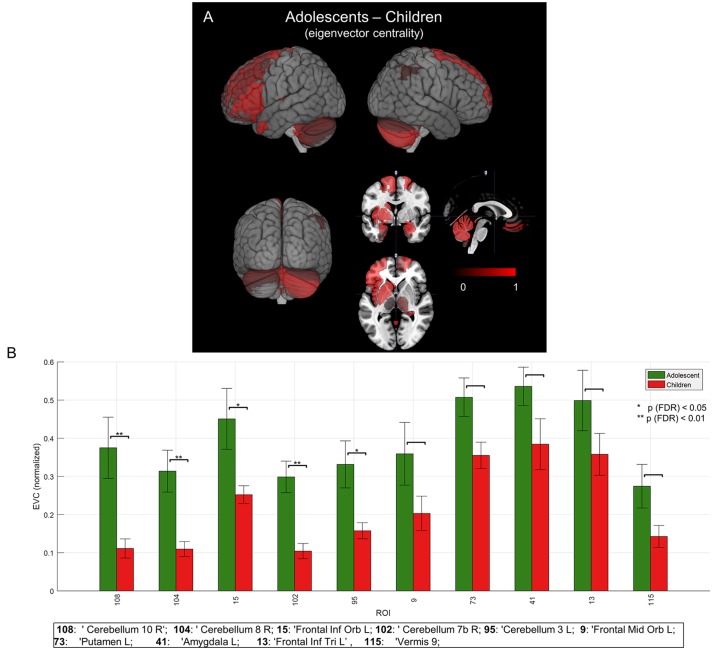
**Mean group network-based parcellation difference between adolescent and children. (A)** Cortical (left inferior frontal gyrus (IFG)) and subcortical (left and right cerebellum) regions detected by group network difference between adolescents and children. **(B)** Regions of interests (ROIs) detected in group differences of adolescents and children. Error bars represent a standard error (SE). “*” and “**” represent *p* (FDR) < 0.05 and < 0.01, respectively.

To evaluate the developmental changes, we compared group network differences between young children and adolescents. As shown in Figures 5A,B and are summarized in the last column of Table 1, significant (*p* < 0.05, FDR corrected) differences were found in right cerebellar regions as well as significant differences in a cortical region (left frontal inferior orbital) and a subcortical region (left putamen in basal ganglia). This may imply a significant role of cerebellar regions in developmental changes from childhood to adolescence (or adulthood), as discussed below.

## Discussion

This is one of the first demonstrations of using large-scale connectivity and graph theoretical (network) analysis to identify hubs associated with language processing. Our results are generally consistent with previous findings in terms of lateralization and localization in early development. Findings provide support for underlying dynamic topological organization of the pediatric language network.

Group source analysis showed low-beta [13, 23] Hz ERD at left inferior frontal and posterior temporal regions, consistent with previous MEG expressive language studies (Singh et al., 2002; Hirata et al., 2004; Kadis et al., 2008, 2011; Ressel et al., 2008; Pang et al., 2011; Doesburg et al., 2012). Whole brain connectivity based on PLV metric derived from broad-band (3–30 Hz) sources suggested prominent connectivity at left perisylvian sites (Figure 1B) and hubs in left prefrontal regions (Figure 1C).

Interestingly, we found stronger hubs in left prefrontal regions of the adolescents than the children (Figure 4 and Table 1), including anterior regions of the IFG (pars orbitalis, or BA47). The developmental increase in left inferior frontal connectivity is consistent with recent studies of effective connectivity patterns in children performing verb generation (Kadis et al., 2015); the localization suggests a pivotal role of the anterior inferior frontal cortex for expressive language, involving regions that extend beyond canonical Broca’s area.

The network analysis for the young children suggested bilateral hubs in cortical and subcortical language regions, but with stronger effects in the right hemisphere (Figure 4A). Such right hemispheric effects were clearly observed in the network contrast of young children against adolescents (Table 1). Previous findings based on changes in cerebral blood flow (dynamic SPECT) have demonstrated a right-dominance in infants and toddlers, with a subsequent shift toward the left after 3 years of age (Chiron et al., 1997). Our results support early right hemispheric effects, (see also Table 1).

Beyond the canonical language regions, our network analyses revealed strong hubs at subcortical regions, including the lentiform nuclei (putamen and globus pallidus), in both children and adolescents (see Figure 4 and Table 1). Findings support the notion that both cortical and subcortical regions are critical for expressive language (Houk, 2005; Booth et al., 2007; Mestres-Missé et al., 2008). Others have indeed shown that subcortical regions, along with cortical-subcortical interactions, may play a critical role in a variety of language processes (Johnson and Ojemann, 2000; Wahl et al., 2008). Group network analyses revealed prominent hubs in the right cerebellum (Figure 4). Significant hubs were found in the same regions for the contrasted network of adolescents against young children (Figure 5). This may imply the importance of right cerebellum in generation of verbs as well as development of the expressive language network (Gabrieli et al., 1998; Middleton and Strick, 2000; Frings et al., 2006; Booth et al., 2007; Houk et al., 2007; Murdoch, 2010; Berl et al., 2014; Verly et al., 2014).

MEG permits recordings on the temporal scale of neuronal oscillations (ms) and fine spatial resolution (mm). A recent retinotopy mapping study has shown that MEG is able to estimate signals ~7 mm in smooth regions of cortex and less than 1 mm for signals near curved gyri (Nasiotis et al., 2016). The high temporal and spatial resolution makes the MEG superior to fMRI for connectivity investigations, i.e., identifying interactions (temporally organized bursts) that occur within milliseconds, over millimeters (Bastos and Schoffelen, 2016). In pediatric studies, MEG is preferable because it offers less restrictive and less noisy testing environment than nMR. Yet, careful considerations are required in connectivity/network estimation; sensor-level connectivity will be inflated by common-source/mixing problems, and source localizations are inherently imperfect (inverse solution, underdetermined). However, the use of source analytic techniques that are robust to noise and correlated/coherent sources, and an understanding of the limitations of both the inversion and connectivity metrics chosen, make MEG network analyses tenable (Schoffelen and Gross, 2009; Bastos and Schoffelen, 2016).

MEG has a lower sensitivity to subcortical sources due to complex cytoarchitecture of brain and the distance from sensors (Hillebrand and Barnes, 2002). Despite this challenge, many previous studies using both simulated and real data have successfully demonstrated estimates of subcortical generators e.g., in hippocampal and basal ganglia (David et al., 2011; Quraan et al., 2011; Mills et al., 2012; for a review see Attal et al., 2012). To improve access to deep neuronal activity, we employed a semi-realistic single shell forward model by Nolte (2003), and a differential beamformer, which is less sensitive to background noise and do not underestimate deep sources (Quraan et al., 2011). Our subcortical findings are also in agreement, from two aspects, with a recent fMRI study on speech control (Fuertinger et al., 2015). First, they demonstrated the usefulness of the graph theoretical analysis in characterizing speech and language control network. Second, they detected communities (hubs) at prefrontal cortex and subcortical (insula, thalamus and putamen) and cerebellum regions. Yet, the subcortical estimates by MEG can be further evaluated using a realistic pseudo-MEG data wherein alternative forward and inverse modeling techniques can be conveniently used (Haufe and Ewald, 2016).

The current study shows that a whole brain connectivity and graph theoretical analysis of MEG data is powerful for characterizing topological properties of the complex language network. The framework is sufficiently general to allow for application to other domain, also.

## Author Contributions

DSK certifies that each of the authors made significant, intellectual contributions to this study and the development of the manuscript. VY conceptualized the methodological framework, and carried out the bulk of analyses for the current study. He prepared the initial manuscript, and carried out revisions after consulting with coauthors. BJW has been studying language models, and the potential for using graph theory metrics for identification of critical sites. BJW advised on graph analyses, choice of metrics and provided edits on the manuscripts during development. The work was carried out in DSK’s laboratory, and was made possible through a grant provided to DSK by the Research Institute at Cincinnati Children’s Hospital Medical Center. DSK oversaw all aspects of the study, from conceptualization, recruitment, imaging, analyses and authorship of this manuscript.

## Conflict of Interest Statement

The authors declare that the research was conducted in the absence of any commercial or financial relationships that could be construed as a potential conflict of interest.
